# Bispecific Antibodies and Other Non-CAR Targeted Therapies and HSCT: Decreased Toxicity for Better Transplant Outcome in Paediatric ALL?

**DOI:** 10.3389/fped.2021.795833

**Published:** 2022-02-02

**Authors:** Krisztián Miklós Kállay, Mattia Algeri, Jochen Buechner, Aviva C. Krauss

**Affiliations:** ^1^Pediatric Hematology and Stem Cell Transplantation Department, National Institute of Hematology and Infectious Diseases, Central Hospital of Southern Pest, Budapest, Hungary; ^2^Department of Pediatric Hematology and Oncology, Scientific Institute for Research and Healthcare (IRCCS), Bambino Gesù Childrens' Hospital, Rome, Italy; ^3^Department of Pediatric Hematology and Oncology, Oslo University Hospital, Oslo, Norway; ^4^Division of Hematopoietic Stem Cell Transplantation, Department of Hematology-Oncology, Schneider Children's Medical Center of Israel, Petach Tikvah, Israel

**Keywords:** blinatumomab, inotuzumab, haematopoietic stem cell transplantation (HSCT), Trisomy 21 (down syndrome), infant ALL, paediatric acute lymphoblastic leukaemia (ALL)

## Abstract

This review will address the place of innovative, non-chemotherapy, non-CAR-T targeted therapies in the treatment of Acute Lymphoblastic Leukaemia (ALL), focusing on their use in the hematopoietic stem cell transplant (HSCT) context. The focus will be on the agent with the most experience to date, namely the bispecific T-cell engater (BiTE) blinatumomab, but references to antibody-drug conjugates (ADCs) such as inotuzumab ozogamicin and monoclonal antibodies such as daratumamab will be made as well. Specific issues to be addressed include: (1) The use of these agents to reduce measurable residual disease (MRD) prior to HSCT and their potential for improved transplant outcomes due to reduced toxicity compared to traditional chemotherapy salvage, as well as potentially increased toxicity with HSCT with particular agents; (2) the appropriate sequencing of innovative therapies, i.e., when to use BiTEs or antibodies versus CARs pre- and/or post-HSCT; this will include also the potential for impact on response of one group of agents on response to the other; (3) the role of these agents particularly in the post-HSCT relapse setting, or as maintenance to prevent relapse in this setting; (4) special populations in which these agents may substitute for traditional chemotherapy during induction or consolidation in patients with predisposing factors for toxicity with traditional therapy (e.g., Trisomy 21, infants), or those who develop infectious complications precluding delivery of full standard-of-care (SOC) chemotherapy during induction/consolidation (e.g., fungal infections); (5) the evidence we have to date regarding the potential for substitution of blinatumomab for some of the standard chemotherapy agents used pre-HSCT in patients without the above risk factors for toxicity, but with high risk disease going into transplant, in an attempt to decrease current rates of transplant-related mortality as well as morbidity; (6) the unique toxicity profile of these agents and concerns regarding particular side effects in the HSCT context. The manuscript will include both the data we have to date regarding the above issues, ongoing studies that are trying to explore them, and suggestions for future studies to further refine our knowledge base.

## Introduction

Relapse of B-cell precursor acute lymphoblastic leukaemia (BCP-ALL) in the paediatric population is relatively uncommon, with an incidence of about 15%. However, children with relapsed disease have a median 5-year survival rate of 25-50% (1). Allogeneic haematopoietic stem cell transplantation (HSCT) is currently the gold-standard treatment for patients with high-risk relapse, as well as for a subset of patients with high-risk primary disease, as chemotherapy alone produces dismal outcomes. Survival after HSCT is highly affected by the remission induction strategy before the procedure. A significant proportion of paediatric patients cannot proceed to HSCT because of serious adverse events from previous therapies, or an inability to achieve an acceptably deep remission with these therapies. Chimeric Antigen Receptor T-cell (CAR-T cell) therapy has yielded promising results in children and adolescents/young adults (AYAs) with relapsed or refractory (R/R) ALL, but carries the challenges of T-cell collection and manufacturing. In contrast, inotuzumab ozogamicin (InO), a humanised monoclonal antibody-drug conjugate (ADC) targeting CD22, can readily be administered. Blinatumomab, a bispecific anti-CD3/anti-CD19 T-cell-engager (BiTE), links the patient's CD3+ T cells to CD19+ blasts, inducing cytotoxicity; it is also available “off the shelf.” The substitution of standard chemotherapy consolidation with non-CAR-T cell immunotherapy promises a new approach to induce deeper remissions with less toxicity compared with current chemotherapy strategies. In this review we summarise the available data regarding these immunotherapy approaches in the paediatric population and try to provide some guidance on choosing between them. A separate review of CAR-T cell therapy in paediatric ALL is provided as a companion paper by Buechner et al. in this Frontiers in Pediatrics supplement.

## Strategies Prior to HSCT

### Blinatumomab Prior to HSCT

The first trial studying systematically the efficacy and safety of blinatumomab in children and adolescents was a phase I/II open-label, single-arm study performed at 26 study sites in Europe and the US (Clinicaltrials.gov identifier; 2, PMID: 27998223). Eligible patients were <18 years of age and had R/R BCP ALL with >25% bone marrow blasts at enrolment. Disease status was primary refractory, patients in first relapse after a full salvage induction regimen, in second or later relapse, or in any relapse after allogeneic HSCT. Forty-nine patients were treated in phase I and 44 patients in phase II. In phase I, the maximum tolerated dose (MTD) of blinatumomab was determined to be 15 μg/m^2^/day. The recommended phase 2 dose (RP2D) was determined as 5 or 15 μg/m^2^/day (1 week of 5 μg/m^2^/day followed by 3 weeks of 15 μg/m^2^/day during the first cycle and for all subsequent cycles). Of the 70 patients treated with the recommended dose, 39% achieved a complete remission (CR) within the first two cycles of blinatumomab, with 52% of the responders achieving complete measurable residual disease (MRD) negativity. Thirteen patients went on to allogeneic HSCT in blinatumomab-induced remission, seven of whom had been transplanted previously. The study showed that blinatumomab had antileukaemic activity and induced remissions across all age groups, including in patients with unfavourable cytogenetics (2).

In the blinatumomab expanded-access program (the RIALTO trial; NCT02187354), patients with a second or later relapse, any relapse after allogeneic HSCT, or who were refractory to other treatments, received blinatumomab for 1–2 induction cycles with the option to receive up to three additional blinatumomab consolidation courses. In total, 110 patients were enrolled. At screening, 11% of all patients had <5% bone marrow blasts, while the remained had ≥5%. Sixty-nine of the 110 study patients (63%) had CR as best response in the first two cycles; of these, 45 (65%) proceeded to HSCT. MRD response was dependent on the pre-infusion blast count, being 47 and 92% for patients with ≥5 or <5% blasts, respectively (3).

Keating and colleagues reported on 15 children (median age 9 years, range 0.5–19 years) with B-cell ALL from five North American paediatric centers who received blinatumomab in CR (10 CR1, 5 CR2) due to persistent MRD [0.01-2.2% by flow cytometry (FCM)] prior to HSCT. Fourteen of the 15 patients had MRD reduced to undetectable levels and were able to proceed to HSCT without significant delay or organ toxicity (4). Overall survival (OS) at 1 year was 93.3% and there was no 100-day treatment-related mortality (TRM), although one patient died past the 100-day mark of chronic graft vs. host disease (cGVHD).

Finally, the North American Children's Oncology Group (COG) designed a randomised trial for children and AYA to more rigorously assess blinatumomab in patients of this age group with a first high-risk B-ALL relapse (5). Enrollment was open from age 1 to 30 years, and 208 patients were included. After receiving re-induction chemotherapy, patients were randomised to either two cycles of blinatumomab or two cycles of multi-agent chemotherapy. The primary endpoint was disease free survival (DFS), with safety and toxicity as secondary objectives. The randomisation was terminated early based upon a data and safety monitoring committee (DSMC) recommendation despite not meeting the stopping rules for efficacy or futility, due to a combination of improved 2-year DFS (54.4% in the blinatumomab arm vs. 39% in the chemotherapy arm), 2-year OS (71.3 vs. 58.4%, respectively) and reduction in MRD at the end of cycle 2 (66 vs. 32%, respectively) with lower adverse event of special interest (AESI) rates in the blinatumomab arm. Moreover, the frequency of infections (15%), febrile neutropenia (5%), sepsis (2%), and mucositis (1%) in the blinatumomab arm were significantly lower compared to the chemotherapy arm (65, 58, 27, and 28%, respectively). Due to premature closure, the study was underpowered for the primary endpoint of DFS (*p* = 0.03); all statistics were descriptive, but as a whole they support the positive benefit: risk assessment regarding the utility of blinatumomab in the treatment of high-risk B-ALL in first relapse.

Locatelli et al. similarly randomised 108 children from age 28 days to 18 years with high-risk B-ALL in first relapse to either one cycle of blinatumomab or one cycle of chemotherapy as the third consolidation element (6). This study was terminated early as well, this time consistent with a stopping rule due to the benefit of blinatumomab. The primary endpoint was event-free survival (EFS). Events were defined as relapse, death, secondary malignancy and failure to achieve CR. The EFS was 66.2% in the blinatumomab arm and 27.1% in the chemotherapy arm (*p* < 0.001). All secondary and exploratory outcomes were in favour of blinatumomab.

In summary, current evidence points toward the efficacy and manageable toxicity of blinatumomab in paediatric patients with BCP-ALL. This is specifically the case in the context of MRD-positive disease prior to HSCT, and as a substitution for single chemotherapy blocks to induce deeper remissions and increase eligibility for subsequent HSCT.

### Inotuzumab Ozogamicin Prior to HSCT

InO is a humanised monoclonal ADC targeting CD22-positive cells; it delivers the potent cytotoxin calicheamicin directly to leukaemic blasts. InO has demonstrated impressive single-agent activity in the adult setting [response rate 78.4 vs. 28.1% *p* < 0.0001; INO-VATE trial, (7)]. However, its efficacy and safety in children are less well-described. The ITCC-059 study [EU Clinical Trials Register (EudraCT) identifier 2016-000227-71] prospectively investigated the RP2D of InO in children aged 1-18 years with R/R CD22-positive ALL (8). Twenty-five patients (including five patients <6 years old) were treated, of whom 23 were evaluable for dose-limiting toxicities (DLTs). The approved dosage for adults (1.8 mg/m^2^ per dose, consisting of 0.8 mg/m^2^ on day 1, 0.5 mg/m^2^ on day 8 and 0.5 mg/m^2^ on day 15) was found to be the RP2D in children as well. Responses included 15 patients (60%) who achieved CR at one of the 2 dose levels studied, 1 patient who achieved a CR with incomplete platelet recovery (CRp), and 4 patients who achieved a CR with incomplete haematologic recovery (CRi); sixteen of the 19 responders for whom MRD data were available achieved MRD-negativity. Three patients treated at the RP2D or the dose level below experienced hepatic DLTs, prompting implementation of a protocol amendment regarding transaminase monitoring and stricter dose delays; no further hepatic DLTs occurred at the RP2D. One patient at experienced prolonged haematologic recovery at the RP2D. Notably, there were no cases of hepatic veno-occlusive disease/sinusoidal obstruction syndrome (VOD/SOS) reported during treatment with InO, nor in the seven patients who proceeded to HSCT after InO therapy. However, two patients treated with InO subsequently experienced VOD/SOS during treatment with multi-agent chemotherapy upon disease relapse.

Brivio and colleagues retrospectively reviewed the data on 15 patients under 3 years of age treated internationally with InO for the same indication. Seven patients achieved CR (47%) and one became MRD-negative after MRD-positivity. The 6-month OS was 47% [95% confidence interval (CI): 27-80%]; two patients developed VOD/SOS after transplant, including one patient for whom this was fatal (9). The authors noted that no specific safety concerns were raised in the two patients <1 year of age upon InO infusion, nor in the four additional patients whose weight was <10 kg at the time of the infusion (9).

Bhojwani et al. reported on 51 children (age 2.2-21.3 years, median 11.5 years) with R/R ALL treated on a paediatric InO compassionate use (CU) programme. Complete responses were seen in 67% of the patients who were treated for overt relapse, and 71% of responders achieved MRD-negativity in the bone marrow, in most patients after the first cycle (10). The administration of InO was initially generally well-tolerated, even by patients who were heavily pre-treated by multiple lines of therapies, and none of the patients developed VOD/SOS during InO therapy. However, 21 patients underwent HSCT after InO with a median time from last dose of InO to stem cell infusion of 26 days. Eleven of these 21 patients (52%) developed post-HSCT VOD/SOS, including 5 in whom this was severe (24%), and 2 in whom it was fatal. The 12-month EFS and OS rates were 23.4 and 36.3%, respectively. A small cohort of patients experienced CD22-negative relapse (3).

Bearing in mind the different treatment contexts, it is noteworthy that in the adult experience of InO, while demonstration of clinical benefit was shown based on durable CR and MRD-negative CR rates in the INO-VATE ALL trial, the analysis of OS did not meet the pre-specified boundary for statistical significance. Additionally, the US Food and Drug Administration (FDA)-approved label for relapsed/refractory adult B-ALL included a boxed-warning for hepatotoxicity, including fatal VOD as well as post-HSCT non-relapse mortality in the InO arm.

In line with the blinatumomab and InO data summarised above, Spanish data in 29 children indicate similar remission rates of 47.6% with either blinatumomab or InO, and reduction of MRD while avoiding further toxic chemotherapy prior to HSCT (11).

In summary, InO is a promising drug that is currently best studied in the setting of residual MRD or refractory disease. With current HSCT strategies, preventive supportive care and close monitoring according to paediatric guidelines, VOD/SOS might well be manageable in children. A systematic and prospective phase II study in children is currently ongoing (ITCC-059, EudraCT: 2016-000227-71), which is investigating InO both as monotherapy and in combination with chemotherapy for high-risk and very-high risk relapsed BCP-ALL in patients ≥ 1 to <18 years of age at the time of enrollment. Another COG study (NCT02981628) is investigating InO in combination with a chemotherapy backbone in patients 1-21 years of age with R/R BCP-ALL. The upcoming IntReALL trial may plan InO as an induction therapy in patients with high-risk relapsed B-ALL.

### Blinatumomab in Combination With Other Targeted Immunotherapy, Prior to HSCT

A recent case report describes an 11-year-old child with primary refractory ALL in whom repeated cycles of blinatumomab and InO allowed achievement of molecular remission, serving as bridging therapy to a successful HSCT (12).

Brethon et al. reported an interesting case report where blinatumomab and gemtuzumab ozogamicin were combined in a 4-month old child with KMT2A-rearranged, mixed-phenotype leukaemia (13). Subsequently, the child was transplanted, relapsed and achieved remission again with CAR T-cell therapy.

## Looking Toward the Future: The Optimal Pre-HSCT Regimen

Novel targeted regimens are evolving in diseases mostly affecting adults, such as chronic lymphocytic laeukemia (CLL), in which combinations of targeted biologic therapies (e.g., a Bruton's tyrosine kinase [BTK]-inhibitor and a monoclonal antibody; or a B-cell lymphoma 2 [BCL2]-inhibitor and a monoclonal antibody) can replace traditional chemotherapy (14). Patients treated with these protocols can enter the transplant unit without a history of sepsis, neutropenic fever, aplasia, organ injury or even alopecia. As our armamentarium of targeted therapies for B-ALL grows, we aim to find context for less toxic therapies for children with this disease as well.

Although the COG study (5) failed to demonstrate a significant improvement in DFS for patients with a first high-risk B-ALL relapse treated with blinatumomab due to premature study closure, this strategy was extremely well-tolerated, reducing many of the complications associated with repeated cycles of chemotherapy. The trial also trended toward higher OS using blinatumomab instead of chemotherapy consolidation. The goal of the treatment strategy in this trial was to bridge to HSCT, and blinatumomab appeared adequate to accomplish this. While the majority of patients became MRD negative after the first cycle, a few patients (10%) became MRD positive after the second cycle. These data support progressing to transplant after the first cycle of blinatumomab in the design of future clinical trials.

Thus, blinatumomab appears to be a highly-promising choice for consolidation therapy before allogeneic HSCT in children and AYAs with a first relapse of B-ALL. Unlike CAR-T cells, blinatumomab is readily available as a pre-manufactured drug, an important advantage in these clinical scenarios, as children with relapse often require immediate treatment. CAR-T cell therapy shows promising results in children with multiply relapsed or refractory disease. However, this therapy has yet to be rigorously evaluated in patients in first relapse or with *de novo* very high-risk disease and compared to other strategies, including blinatumomab and InO. Moreover, it necessitates patient-specific manufacturing processes that can take precious time.

InO is an off-the-shelf drug. It is convenient to administer as a short intravenous infusion in contrast to the continuous 28-day infusion of blinatumomab, and therefore it can be combined with other therapies, as is currently investigated in the paediatric ITCC-059 study. However, the risk of developing VOD/SOS during subsequent treatment warrants further investigation in the pre-HSCT setting, especially in children.

To date, there are insufficient data directly comparing the various non-chemotherapeutic strategies prior to HSCT to decisively clarify whether blinatumomab, InO or even CAR-T cell therapy are the optimal pre-HSCT therapy. Specifically, the decision to reserve the use of CARs for post-HSCT relapse or to use them in the relapse setting to achieve remission prior to allogeneic HSCT is a subject of considerable debate in the paediatric laeukemia community. Factors influencing the decision whether or not to consolidate CAR-T cell therapy with HSCT are discussed in detail in the companion paper by Buechner and colleagues in this same Frontiers in Paediatrics supplement. Of note, if CAR-T is being used solely as a “bridge” to HSCT- to induce remission pre-HSCT rather than as definitive therapy- it remains to be seen whether blinatumomab, given the advantages discussed above, might be a more appropriate choice.

The administration of blinatumomab in CR1, as a substitute to standard chemotherapy or even bridging to transplant is currently being evaluated in several trials (NCT03914625, NCT04604691, NCT05029531). Extrapolating data from the trials in ALL in first relapse predicts a high likelihood that blinatumomab can find its place in this setting as well, potentially lowering MRD with less associated toxicity and possibly improving HSCT outcomes in this population.

## Special Populations

While mainly studied thus far in the R/R population, ongoing studies have capitalised on the relatively decreased toxicity profile seen with blinatumomab to explore its role in the treatment of subpopulations of patients with ALL for whom standard chemotherapy is particularly toxic. Two specific historic subpopulations are patients with Trisomy 21 (Down syndrome, DS) and infants, although patients who develop infectious complications that may interfere with their ability to tolerate standard chemotherapy are relevant candidates as well.

### Patients With Down Syndrome

In addition to a higher risk of relapse, patients with DS have been shown to have an increased risk of treatment-related mortality with traditional induction and consolidation chemotherapy (15, 16), such that they have heretofore often been excluded from clinical trials in ALL or have received adapted treatment elements with reduced intensity. Interestingly, although numbers are limited, the data on transplantation in patients with DS and ALL suggest that the main obstacle these patients face is relapse rather than TRM post-HSCT (17). Therefore, patients with DS appear to be an ideal population to study targeted agents with the potential to reduce toxicity without sacrificing efficacy.

The data to date on the use of blinatumomab in patients with DS is limited to mostly case reports (18–20). At one of our centres we have utilised blinatumomab post-induction in a 6-year old patient with DS who developed vocal cord paralysis with vincristine, requiring prolonged intubation and ultimately tracheostomy; blinatumomab treatment allowed her to achieve a CR, which was consolidated with a matched-sibling HSCT over 9 months ago. Most ongoing studies investigating blinatumomab either up-front or at relapse are including patients with DS. Specifically, the randomised COG study AALL1731 (NCT03914625), explores the incorporation of blinatumomab into the post-induction phases of treatment for patients with protocol-defined standard-risk ALL that is average or high-risk (based on cytogenetic, molecular and other features) and who have reached an MRD level of <0.1 by the end of consolidation. AALL1731 allows for the inclusion of patients with DS; specifically, those who are standard risk, without high-risk features, and below a threshold MRD level at the end of induction may be randomised, as are patients with standard-risk ALL without DS, to receive post-consolidation therapy with or without blinatumomab. Patients with DS whose MRD at the end of induction is above the threshold required for randomisation have blinatumomab incorporated into post-consolidation therapy. In addition, the ALLTogether1 protocol (NCT04307576), which comprises both randomised and non-randomised interventions for various risk-strata in patients aged 1-45 years of age with newly-diagnosed ALL, includes an arm for patients with DS, in which standard “Consolidation 1” and “Consolidation 2” are replaced with blinatumomab. The primary endpoint for this study is EFS.

While it is premature to make any predictions regarding the safety or efficacy of blinatumomab in patients with DS, the results of these studies will inform decision-making for this cohort of patients and give a more objective answer as to whether the use of blinatumomab has the potential to improve their outcomes by decreasing treatment-related mortality without compromising efficacy. These studies will also provide a more granular toxicity profile for patients with DS who are treated with these agents, which could potentially allow for risk-mitigation strategies that will further enhance the safety of their use in this fragile population. Finally, if the use of blinatumomab in patients with DS is in fact shown to allow for an increased number of these patients to achieve an MRD-negative state with less toxicity than traditional chemotherapy, this could potentially affect the HSCT outcome of these patients as well, given the data referred to above (17) suggesting that relapse is the primary obstacle when patients with DS undergo HSCT.

### Infant ALL

ALL in patients under 1 year of age, so-called “infant ALL,” has a particularly poor prognosis, especially for the approximately 75% of patients who have a KMT2A rearrangement, for whom the expected 5-year EFS is as low as 35% (21–23). Even patients without a KMT2A-rearrangement have outcomes that are poorer than those seen in children with ALL overall, with EFS as low as 60% at 5-years using the COG protocol (22), although more recent studies have shown 6-year EFS as high as 73% in these patients (23). As such, induction for infants with ALL is generally uniform regardless of cooperative group or region, and includes prednisone followed by dexamethasone, as well as the use of anthracyclines and the standard induction agents. In contrast, the decision to proceed with HSCT in infants with ALL varies, mostly being reserved for those with highest-risk disease (21). The role of HSCT in infant ALL is further discussed in the companion paper by Bierings and colleagues in this supplement.

In the recently published Interfant-06 protocol [NCT00550992; (23)], infants with newly-diagnosed ALL were defined as low-risk (LR) if they were KMT2A-wild type, high-risk (HR) if they had a KMT2A-rearrangement and were older than 6 months with a white blood cell (WBC) count of ≥300 x 10^9^/L, or had a poor prednisone response, and medium-risk (MR) if they had a KMT2A-rearrangment without the other high-risk features. The protocol randomised MR and HR patients to a course of post-induction “lymphoid” therapy (Protocol IB of the standard ALL protocols; *n* = 161 patients), or two courses of post-induction “myeloid” therapy [cytarabine/daunorubicin/etoposide (ADE) and mitoxantrone/cytarabine/etoposide (MAE); *n* = 169 patients]. Patients in all risk-categories proceeded to two further courses of identical therapy before entering maintenance, with the exception of those proceeding to HSCT, who did so after the first identical cycle. Criteria for HSCT were all HR patients, and during the course of the study, this was extended as well as to MR patients with an MRD of ≥10^−4^ at the end of that cycle. The study had 80% power to detect a DFS difference of 16% at 3 years, assuming 41% DFS in the control arm, with an alpha of 0.05; the study failed to show a difference in DFS between the randomised arms, with 4- and 6-year DFS of 42.2% [standard error (SE) 3.9] and 39.3% (SE 4), respectively, in the “myeloid treatment” arm, and 37.8% (SE 3.9) and 36.8% (SE 3.9) in the “lymphoid treatment” arm. OS at 6-years was similar in the two arms, being 54.4% (SE 4.0) in the “myeloid” arm and 47.1% (SE 4.2) in the “lymphoid” arm, with a nominal *p*-value of 0.2706. Finally, cumulative incidence of relapse (CIR) at 6-years was similar between the arms (47.5%; SE 4.0 vs. 54.9%; SE 4.1), as were the rates of deaths in continuous complete remission (CCR; 10.2%; SE 2.4 vs. 8.3%; SE 2.2). However, relevant to the focus of this review, there was deviation from randomisation and outcomes for those patients who underwent HSCT in this study. Specifically, although all HR patients were to proceed to HSCT in CR1 after cycle 3 (lymphoid arm) or 4 (myeloid arm), only 76 of the 143 patients in the HR subgroup actually proceeded to transplant in CR1, due to earlier events in the 54 remaining patients, mostly relapses (numbers not specified). For the 76 patients who proceeded to HSCT, the 4-year DFS was only 44%, comprising 26 (34.2%) patients who relapsed, 14 (18.4%) who died in CR due to HSCT-related toxicity, and two patients who developed a second malignancy. Once the protocol was amended to include HSCT recommendations for MR patients who did not achieve MRD-negativity, only 16 of the 23 patients who met these criteria proceeded to HSCT, and the 4-year DFS in this cohort was only 18.8%. It is notable that the death rate in CR post-HSCT dropped from 26% (*N* = 50) in those undergoing HSCT between 2006 and 2011, who received busulfan, cyclophosphamide and melphalan conditioning, to 5% (*N* = 61), in those undergoing HSCT with busulfan, fludarabine, treosulfan, and thiotepa conditioning. Interestingly, in the patients treated with “myeloid” therapy, the death rate in CR was similar overall (10.1%) to that of those treated with “lymphoid” therapy (8.1%), but more of these deaths were considered related to HSCT in the latter (5%) than in the former (3%). This suggests that intensive therapy followed by HSCT in these patients carries with it a not-insignificant risk of mortality that may be related to the intensity of therapy required in these patients.

Although *post-hoc* analyses are limited, the lack of improvement in outcomes, the limitations of HSCT in either treatment arm, and the high number of relapses and toxic-deaths in this large study of patients with infant ALL, raises the question as to whether in this population, less-intensive, targeted therapy might improve outcomes overall and potentially also outcomes with HSCT in relevant subpopulation(s). This is the subject of a current pilot study (see below).

Experience with blinatumomab in infants is limited. The largest cohort of patients reported to date was a retrospective study done in the UK and the Republic of Ireland (24). They detailed the treatment of 11 patients with infant ALL treated between 2016 and 2019, after treatment per the Interfant-06 protocol, with blinatumomab either for persistent MRD or for disease relapse. One patient was over 1 year at the time of treatment for blinatumomab; the remainder ranged from 0.4 to 0.75 years old. Two patients received blinatumomab in CR1, 6 in CR2, 2 with primary refractory disease, and 1 in first relapse. Most patients received one cycle of blinatumomab, while two patients received two cycles. Of the 10 patients aged <1 year described in the paper, MRD ranged from 0.06 to 9% prior to treatment. In two patients with 9 and 0.3% MRD prior to blinatumomab therapy, MRD was 0.05 and 0.06%, respectively, following blinatumomab therapy. In the other seven patients, MRD was <0.005% after treatment with blinatumomab, with one patient receiving two cycles. One patient had grade 2 cytokine-release syndrome (CRS) and 2 had grade 1 CRS. The patient with grade 2 CRS also experienced neurotoxicity (somnolence and confusion) that required treatment interruption and dose reduction, which was tolerated upon re-challenge. All patients proceeded to HSCT after blinatumomab therapy; the median follow-up for all patients post-HSCT was 267 days (range 58-1,163). Notably, of four patients who relapsed post-HSCT, three relapsed with CD19+ disease and were able to receive CART therapy which induced another CR; one patient experienced a lineage switch to acute myeloid leukaemia (AML). One patient's death was attributed to transplant-related mortality, and at the time of publication, 3-year EFS for the cohort was 47% and OS was 81%, although due to its retrospective, non-randomised nature, comparison of outcomes to historical cohorts treated with traditional chemotherapy is subject to the usual limitations. The authors concluded that blinatumomab can be safely administered in this young age group, and was able to induce molecular remission in a majority of patients, allowing consolidation with HSCT, although they acknowledged the limitations of their small sample size.

Other case reports with even smaller numbers of patients yield similar outcomes to the study outlined above (13).

To allow for a more comprehensive assessment of the safety and efficacy of blinatumomab in infant ALL, the goal of the ongoing Interfant pilot study, Trial NL5993 (Netherlands Trial Register identifier: NTR6359) is to test the feasibility of adding blinatumomab to the Interfant-06 protocol. The group states that “the toxicity and safety data of this pilot study will directly influence the drug choice and schedule given to infants in the worldwide collaborative COG/JPLSG/Interfant group trial” (Netherlands trial register website). Of note, inclusion criteria require that patients enrolled in this study are in CR post-induction (25); since induction failure is not the major reason for treatment failure in this population, it does not appear that this bias will preclude interpretability of the results of this study regarding the toxicity and outcomes for patients with infant ALL treated with blinatumomab. One issue that has been raised is whether CD19 is the appropriate target in this population (21), and this question can only be answered by prospective studies described above.

### Opportunistic Infections Precluding Standard Chemotherapy

A less well-defined niche in which blinatumomab may be especially suitable is on a case-by-case basis for patients in whom opportunistic infection or other organ toxicities preclude the use of standard chemotherapy. Because this population is not rigidly-defined, the data in these clinical contexts are limited to specific cases within a series or case reports, and the literature is scattered with accounts of patients (often in the R/R setting) in whom blinatumomab was administered to allow for disease control in the face of potentially fatal invasive fungal infections (IFI), including sinus and orbital zygomyces infection and pulmonary fungal infection (26). At one of our centres, we have successfully used blinatumomab as consolidation for a patient with face-distorting and cerebral mucormycosis who were treated radically with surgery and antifungals. In a series by Contreras et al. (27), 2 of 27 patients with B-cell ALL treated with commerical blinatumomab, outside the context of a clinical trial, between 2010 and 2018, were treated in MRD-negative remission to allow parallel delivery of aggressive anti-fungal treatment alongside non-myelosuppressive, anti-leukaemic therapy. As the use of blinatumomab and other targeted therapies becomes more common, the collection and analysis of real-world data (28, 29) will allow a more comprehensive understanding of the role of these therapies in patients with infections that preclude or delay the use of conventional chemotherapeutic agents.

## Blinatumomab and INO in the Post-HSCT Setting

During the last decade, several groups have investigated the prognostic impact of post-transplant MRD in paediatric ALL; MRD after HSCT is a dynamic process and variations of MRD over time are important in predicting outcome. While high levels of post-transplant MRD positivity are strongly predictive of disease recurrence, low level MRD values, especially if detected early after HSCT, are not invariably associated with relapse (30). By contrast, the further the patient is from HSCT, the more likely it is that even low levels of MRD will predict a poor prognosis (30–33). In particular, long-term outcome is excellent not only for those patients who remain MRD-negative, but also for those who achieve MRD-negativity (after an early low-level positivity) at late time-points after HSCT (32, 34). In a recent multicentre study, Bader et al. analysed the relative risk of pre- and post-HSCT MRD in paediatric ALL, showing that, when the two measures were simultaneously evaluated, post-HSCT MRD was more important in determining relapse risk compared with pre-HSCT MRD (34). For patients with detectable post-transplant MRD, the outcome may be influenced by additional factors, particularly by the occurrence of GvHD, supporting the assumption that low levels of residual leukaemic cells can be controlled by an immune-mediated Graft-vs.-Leukaemia (GvL) effect (34). Thus, the main approaches to tackle MRD-recurrence in the post-transplant period have focused on strategies to induce the development of a GvL effect, such as rapid discontinuation (or abrupt cessation) of immune suppression (35–38) and infusion of donor derived lymphocytes or cytokine-stimulated immune effector cells (39, 40). However, with such approaches, the benefit derived from GvL may be offset by the increased TRM associated with severe GvHD, and caution should be used when adopting interventions that stimulate excessive GvHD.

There is thus great interest in the application of blinatumomab and InO to eliminate detectable MRD following HSCT patients with BCP-ALL, in an attempt to prevent overt disease relapse. Furthermore, these approaches are particularly attractive as maintenance therapy, irrespective of MRD-results, for patients with disease deemed at high-risk of relapse, such as those with pre-HSCT MRD positivity or unfavourable cytogenetic features. Many groups are investigating the use of blinatumomab post-HSCT to consolidate remission status.

In the ALL SCTped 2012 For Omitting Radiation Under Majority Age (FORUM) Add-on Study, paediatric patients who are MRD-positive before HSCT or who become MRD-positive after HSCT are candidates to receive blinatumomab after tapering/discontinuation of immune suppression (NCT04785547). The University of British Columbia is conducting a trial in children and adults with B-cell ALL based on sequential post-transplant MRD-testing followed by blinatumomab administration in case of detectable MRD (NCT04044560). The Medical College of Wisconsin is evaluating blinatumomab in children and AYAs with high-risk B-ALL in two different experimental arms: patients who are MRD-negative by flow cytometry (FCM) and high-throughput sequencing (HTS) before transplant will receive reduced-intensity conditioning, while patients with with MRD-negativity by FCM but MRD-positivity by HTS will undergo myeloablative, TBI-based conditioning. All subjects will receive a T-cell receptor (TCR) α/β T-cell- and B-cell-depleted HSCT followed by blinatumomab continuous infusion starting from day 100 after-HSCT (NCT04746209).

The MD Anderson Cancer Center is investigating the use of blinatumomab as a maintenance strategy following allogeneic HSCT in children and adults (NCT02807883). Preliminary results in adults with high-risk B ALL have shown that blinatumomab started within 3 months post-HSCT is well-tolerated. Among the 12 patients treated, none of the 8 subjects with MRD negativity before treatment initiation has relapsed. By contrast, all subjects with positive post-transplant MRD progressed to overt disease recurrence. Of note, the 4 patients who relapsed had a lower CD8/CD4 ratio and higher expression of checkpoint proteins and molecules [particularly programmed death 1 (PD1) and T-cell immunoreceptor with Ig and ITIM domains (TIGIT)] compared to non-progressors (41).

### Potential Post-HSCT Toxicities, and the “Right” Immunologic Milieu?

In the context of allogeneic HSCT, it has been hypothesised that blinatumomab could potentially induce a broader GvL effect by inducing polyclonal donor T-cells expansion, reactivating donor memory-T cells and suppressing B regulatory cells (42, 43). This raises concerns regarding an increased risk for GvHD when blinatumomab is administered in the post-HSCT setting. However, in adult patients who received blinatumomab for B-cell ALL relapse after allogeneic HSCT, GvHD was observed in 11% of cases; the majority of cases were of mild or moderate severity, and did not require blinatumomab discontinuation. Only 2 out of 19 patients with a history of GvHD experienced GvHD reactivation during treatment (44). Similarly, in a cohort of 28 paediatric patients who received blinatumomab after HSCT, no signs of GvHD were recorded (45).

Early administration of blinatumomab for detectable MRD after transplant has the advantage of exploiting the anti-leukaemic effect of blinatumomab in the context of low disease burden, thought to be associated with increased response rates (2), and T-cells of donor origin that have- in contrast to recipient T-cells prior to HSCT- not been exposed to chemotherapy. However, incomplete immune recovery after transplant may negatively affect the efficacy of blinatumomab. Indeed, although it has been previously reported that there is no correlation between response to blinatumomab therapy and absolute numbers of total T cells, higher percentages of CD3+ T-cells and of CD45+ CD3+ CD8+ T cells are associated with a great likelihood of MRD negativity and haematologic remission, respectively, following blinatumomab administration in the adult setting (2, 45, 46). The combination of donor-lymphocyte infusion (DLI) and blinatumomab administration has been proposed as a possible strategy to increase the anti-laeukemic activity of both therapeutic measures and overcome limitations related to partial T-cell reconstitution after transplant. Isolated reports suggest that this approach is safe and effective in adult patients (42, 47), and several groups are investigating this combination in clinical trials in children and adults (NCT03982992, NCT03849651). In order to limit the risk of severe GvHD associated with haploidentical DLI (48), infusion of CD45RA-depleted T cells following a TCRαβ depleted graft, and subsequent blinatumomab administration, is currently under investigation (NCT03849651). One of the main concerns regarding prophylactic blinatumomab administration after HSCT is related to the risk of inducing a loss of target CD19 expression on leukaemic blasts, which would preclude potential future benefit from CD19-directed CARTs (49). Presence of low leukaemia burden should theoretically reduce the risk of stochastic emergence of CD19-negative clones that could escape T-cell immunosurveillance. Despite that, previous exposure to blinatumomab has been associated with a significant higher risk of failure or relapse after CAR-T cell therapy, and shorter survival (50–52).

Like blinatumomab, pre-emptive administration of InO is also under investigation as a strategy to reduce leukaemia relapse after transplantation in both children (NCT03913559) and adults (NCT03104491, NCT03856216). As reviewed above, of particular concern using InO after HSCT is the potential for increased VOD/SOS risk [Brivio et al., 2021, (10)]. Despite that, in a preliminary report of 8 adult subjects with high-risk B-ALL who receive pre-emptive InO administration starting from 40 to 100 days after transplant, no cases of VOD/SOS were observed (53). Similarly, in another study describing the combination of InO and escalating doses of DLI in 8 adults with B-ALL who relapsed after allogeneic HSCT, no patients experienced VOD/SOS. Of note, six out of eight patients treated with this approach obtained MRD negativity after the 2nd course of InO, which was long-lasting in 4 of them (54). Thrombocytopenia is another known toxicity which may limit the application of InO in the post-transplant setting, especially for those patients experiencing delayed platelet recovery (53).

In conclusion, available data are scarce and do not allow one to draw any definitive conclusions regarding the role of pre-emptive blinatumomab or InO administration after HSCT. Although prophylactic immunotherapy is an intriguing strategy to optimise the outcome of HSCT in B-ALL, results of ongoing clinical trials, preferably those that include prospective monitoring of pre- and post-transplant MRD, are much awaited to clarify the efficacy and potential drawbacks of each strategy and to better identify those patients who are likely to most benefit from these approaches.

## Discussion

Table 1 summarises the knowns and unknowns with regard to blinatumomab and InO in the HSCT context. Both therapies have shown safety and efficacy in the treatment of R/R BCP-ALL in children, and show promise as consolidation therapy prior to allogeneic HSCT instead of the standard chemotherapeutic options. These therapies have advantages over CAR-T cell products with regard to universal availability and manufacturing, and rapid access. They may be particularly relevant in populations for whom toxicity is a major obstacle of current bridges to transplant, such as those with DS, infant ALL, or with serious opportunistic infections.

**Table 1:** Knowns and unknowns with regard to various alternatives to traditional cytotoxic chemotherapy before or after HSCT for paediatric BCP-ALL.

	**Knowns**	**Unknowns**	**Ongoing trials**
**Specific agents**			
Blinatumomab	First relapse: improved outcome, decreased toxicity	Specific effects on HSCT outcome	
		Should it be integrated first-line in specific disease subsets?	Interfant—pilot and future protocols AALL1731, various populations, including standard risk patients with Trisomy 21 (NCT03914625) NCT04604691 ALL Together 1, patients with Trisomy 21
		Comprehensive safety/efficacy in infants	Trial NL5993 NCT05029531
Inotuzumab Ozogamicin	Activity in paediatric patients with r/r CD22+ ALL Increased VOD/SOS in adults	Increased VOD/SOS affecting HSCT outcome in children	ITCC-059 (EudraCT Number 2016-000227-71)
**General unanswered questions (unknowns)**			
Use of these therapies in the post-HSCT setting	use as maintenance in HR populations?		Blinatumomab NCT04785547
	impact on GVHD and other HSCT-specific morbidities (e.g., VOD/SOS)?		NCT04044560 NCT047462069
	effect of incomplete immune reconstitution on the efficacy of these therapies?		NCT02807883 NCT03982992 NCT03849651 NCT03849651 InO NCT03913559 NCT03104491 NCT03856216

Some of the critical unanswered questions with regard to blinatumomab and InO pertain to the presumed lower toxicity of these classes of agents in comparison to traditional chemotherapeutic agents. While caution must be exercised when comparing even therapeutics with similar mechanisms in different diseases, in general the InO story is vaguely reminiscent of the history of gemtuzumab ozogamycin (GO), an anti-CD33 targeting ADC linked to calcheamicin. While initially approved in 2000 for the treatment of older patients with relapsed AML, both lack of confirmation of clinical benefit as well as safety concerns, including treatment-related mortality (induction deaths) and VOD/SOS, were associated with its market withdrawal 10 years later (55). Extensive pharmacokinetic (PK) analyses of new dosing regimens as well as updated efficacy and safety data using these regimens led to its approval in 2017 for the treatment of R/R CD33-positive AML in paediatric and adult patients, as well as in combination with the standard “7 + 3” regimen for the treatment of newly-diagnosed CD33-positive AML in adults (56).

The results of ongoing studies will be crucial to inform decision-making in this arena, in particular whether these therapies can produce improved efficacy when given prior to allogeneic HSCT without untoward toxicity, such as VOD/SOS or GVHD, which will lead to enhanced EFS and OS in the long run. Until further, extensive data in children and adults are available, the potential for unique severe toxicities from these therapies, as well as the potential for improved efficacy with their use, should inform the risk-benefit calculus when making treatment decisions between InO, blinatumomab, and CAR-T cell therapies. Finally, we look forward to results of ongoing studies in the post-HSCT application of these therapies in the maintenance or relapse settings to appraise their relevance and potential in improving outcomes for paediatric patients undergoing HSCT for ALL.

## Author Contributions

KK, MA, JB, and AK contributed to conception and design of the analysis. KK, MA, and AK wrote sections of the manuscript. All authors contributed to manuscript revision, read, and approved the submitted version.

## Conflict of Interest

Author JB has received personal fees, advisory board/steering committee honoraria, and non-financial support from Novartis; and advisory board honoraria from Pfizer, Kite, and Janssen. The remaining authors declare that the research was conducted in the absence of any commercial or financial relationships that could be construed as a potential conflict of interest.

## Publisher's Note

All claims expressed in this article are solely those of the authors and do not necessarily represent those of their affiliated organizations, or those of the publisher, the editors and the reviewers. Any product that may be evaluated in this article, or claim that may be made by its manufacturer, is not guaranteed or endorsed by the publisher.
